# Congenital Isolated ACTH Deficiency Caused by *TBX19* Gene Mutation: A Family Report

**DOI:** 10.3389/fped.2019.00546

**Published:** 2020-01-10

**Authors:** Cheng Peng, Guoyu Sun, Zezhong Tang, Xinlin Hou

**Affiliations:** Department of Neonatal Ward, Peking University First Hospital, Beijing, China

**Keywords:** ACTH, CIAD, TBX19, hypoglycemia, seizure, cholestasis

## Abstract

Congenital isolated adrenocorticotropic hormone deficiency (CIAD) is a rare disorder that may be conducive to hypoglycemia, cholestasis, and seizures. We reported on two siblings with a homozygous mutation of the *TBX19* gene, C.377 (exon2) C>T, p. P126L. Their parents had heterozygous mutations on the same locus. Glucocorticoid supplementary therapy was effective, but the treatment became delayed due to inaccessibility, which resulted in entirely different clinical outcomes for the siblings. The older brother developed subdural hematoma, intractable epilepsy, and developmental delays. In contrast, the younger sister received timely glucocorticoid replacement therapy and had no long-term complications while maintaining a good quality of life. In summary, when CIAD is confirmed, early intervention is essential to achieve the optimal outcome.

## Background

Adrenocorticotropic hormone (ACTH) is a key hormone in the hypothalamic-pituitary-adrenal axis. Isolated ACTH deficiency is a rare disease, which presents itself as adrenal deficiency. The clinical classifications are adult isolated ACTH deficiency (AIAD) and congenital isolated ACTH deficiency (CIAD). AIAD commonly occurs in middle-aged and elderly people. The disease is associated with the generation of autoimmune antibodies, which destroys pituitary corticotropin cells (1). CIAD is a rare genetic disorder with clinical and genetic heterogeneity and familial inheritance. This disease may lead to adverse outcomes, or even death, without prompt treatment. Laboratory tests usually show low levels of ACTH and cortisol.

In the current report, an infant female and her older brother suffered from isolated ACTH deficiency caused by a *TBX19* gene mutation. Treatment response in these siblings confirm the necessity of earlier detection and treatment of this disease. Delayed treatment substantially alters the prognosis.

## Case 1

A 52-days-old infant was hospitalized due to a long period of jaundice, a cough lasting 2 days, and (most seriously) severe hypoglycemia commencing a half-day prior.

The patient developed jaundice 2 days after birth, and laboratory tests showed total serum bilirubin (TSB) was 134.6 μmol/L and direct serum bilirubin (DSB) was 15.5 μmol/L. Phototherapy was initiated, however, jaundice recurred 1-week later, and the patient developed acholic stool and dark urine. Maximum TSB was 358.5 μmol/L (21.0 mg/dL) and maximum DSB was 27.1 μmol/L (1.6 mg/dL). The second round of phototherapy resulted in slight improvement, and TSB was reduced to 278.2 μmol/L (16.3 mg/dL). Two days prior to hospitalization, the patient developed a cough and the jaundice worsened. The infant was brought to our outpatient department, and a complete blood count was ordered. The white blood cell (WBC) count was 8.27 ×10^9^/L, revealing lymphocyte predominance (71.8%), and C-reactive protein (CRP) level of 9 mg/L (reference range 0–8 mg/L). Blood biochemistry revealed increased TSB (278.2 μmol/L; 16.3 mg/dL), and suggested liver damage, with an alanine aminotransferase (ALT) level of 120 IU/L (reference range 9–50 IU/L) and aspartate transaminase (AST) level of 422 IU/L (reference range 15–40 IU/L). Surprisingly, DSB was only elevated to 80.3 μmol/L (4.7 mg/dL). To determine the cause of the disease, the infant was referred for an abdominal ultrasound. The fasting infant appeared listless, without seizure activity. In addition, blood glucose was 0.7 mmol/L. The infant was administered a glucose solution and improved. A retest showed 7 mmol/L blood glucose. Soon afterward, the infant was transferred to the neonatal ward for further treatment.

On admission, the infant showed marked cholestasis, hence we administered compound glycyrrhizin, reduced glutathione, polyene phosphatidylcholine, ademetionine, and ursodesoxycholic acid, to provide a comprehensive liver protecting regimen. Albumin and Vitamin K were added for supportive therapy. However, DSB progressively increased to a maximum of 118.8 μmol/L. The patient had high levels of cytomegalovirus-DNA (CMV-DNA) in urine (526 copies/mL) and breast milk from her mother (2,770 copies/mL). These results, in combination with a chest X-ray revealing pneumonia, commenced anti-infective therapy. We performed a bronchoalveolar lavage and found elevated copy numbers of CMV in the alveolar lavage fluid, 2,540 copies/mL. Ganciclovir treatment was added to the regime and the patient was temporarily transferred to the Capital Institute of Pediatrics (Beijing, China) for biliary imaging, gallbladder fistula irrigation, and a liver biopsy. The irrigation was not as effective as expected, further confirming the diagnosis of cholestasis, while the liver pathology indicated CMV-induced hepatitis.

During hospitalization, we suspected an endocrine disorder contributing to the clinical manifestations. The patient suffered from a seizure 4 days after the irrigation, which presented as a loss of consciousness with both eyes gazing to the left, and blood glucose was 2.2 mmol/L. Intravenous administration of glucose was commenced, and the patient recovered to normal in a short time. This hypoglycemic seizure occurred twice while the patient was receiving treatment in the hospital. The electrolyte disturbance was mainly reflected in hyponatremia. Minimum serum sodium fluctuated from 124 to 137 mmol/L. Meanwhile, the endocrine laboratory evaluation indicated a low cortisol and ACTH concentrations. The plasma cortisol level was 0.23 μg/dL (the normal range: 4.4–19.9 μg/dL) and the ACTH level was <1 pg/mL (the normal range: 7.2–63.3 pg/mL). Thyroid-stimulating hormone was high, 16.39 uIU/mL (the normal reference range: 0.66–6.29 uIU/ml), and the patient was given 12.5 μg levothyroxine sodium every other day. We also examined growth hormone (2.56 ng/mL), fasting insulin (8.28 μIU/mL; the corresponding peripheral blood glucose was 7.4 mmol/L) and insulin-like growth factor-1 (35.6 ng/mL). All were within normal ranges.

After a systematic analysis of the patient's clinical features and family history, we suspected CIAD and administered 15 mg/m^2^/day hydrocortisone. Follow-up testing showed: glucose (4.8–6.4 mmol/L), serum sodium (128–135 mmol/L), and TSH 2.2 uIU/mL. No convulsive attacks were observed after the regulation of medication, and the patient was discharged and follow-up was recommended. We confirmed our diagnosis with second-generation sequencing, and the gene analysis is presented below.

Currently, the patient is ~2 years old and receiving long-term oral hydrocortisone treatment. She has not suffered epilepsy nor jaundice since the therapeutic intervention. A developmental evaluation of the patient was performed, and the positive results confirmed the importance of early and active intervention. The patient's Gesell Developmental Schedules results (development quotient) were: Adaptive 91.4, Fine motor 94.7, Gross motor 91.8, Language 89.9, and Personal-social 94.7. The intelligence assessment was within the normal range.

## Case 2

The infant's 5-years-old brother suffered from severe hypoglycemia (1.1 mmol/L) and hyponatremia (126 mmol/L) shortly after birth, and developed cholestasis. The simultaneous TSB and DSB were 178.4 μmol/L (10.4 mg/dL) and 13.7 μmol/L (0.8 mg/dL), respectively. He received an intravenous glucose and hydrocortisone infusion (17 mg, two times per day) for 2 days. He was discharged when his serum glucose returned to normal and jaundice was ameliorated. Within the next few days, the patient became drowsy and had several seizures. The infant was hospitalized again and, on this occasion, presented with jaundice. The blood biochemistry data showed that TSB was 232 μmol/L (13.6 mg/dL) and DSB was 15.9 μmol/L (0.93 mg/dL). The patient received phototherapy and frequent breastfeeding. As before, his serum glucose briefly normalized.

At 2 months of age, the patient suffered from a tonic seizure and was readmitted to the hospital. At this time, his serum ACTH levels were <1 pg/mL and his cortisol levels were significantly reduced. Simultaneous insulin, C-peptide and metabolic screening tests were all normal. Oral dexamethasone therapy (0.75 mg per day) was administered for 3 weeks. The patient went home after glucose levels returned to normal and seizures were under control. Soon after, the patient caught pneumonia, which induced another convulsion, although glucose levels remained normal. The medication was adjusted and oral hydrocortisone (2.5 mg, three times per day, 24 mg/m^2^) was administered for maintenance therapy. The liver dysfunction resolved gradually. The brain MRI revealed a bilateral subdural hematoma, which worsened the seizures. We administered topiramate, but the seizures could not be effectively controlled. After subdural paracentesis for draining the hematoma 4 times and a 4-weeks-period of ACTH treatment, we continued administering hydrocortisone (3.3 mg, three times per day, 11 mg/m^2^) and added levetiracetam + topiramate for antiepileptic therapy. The epilepsy was partially controlled. The frequency was ~7–8 times per year. The elder brother's psychomotor development was delayed. When the patient was 7 years of age, his scores on the Wechsler Intelligence Scale were: Verbal Comprehension Index, 62; Perceptual Reasoning Index, 67; Full-Scale IQ, 60.

## Gene Analysis

Interestingly, both patients experienced the triad of hypoglycemia, cholestasis, and seizures. These clinical manifestations, and the treatment course that followed, gave us clues that the brother and sister may have been suffering from the same, or similar disease.

**Figure 1:**
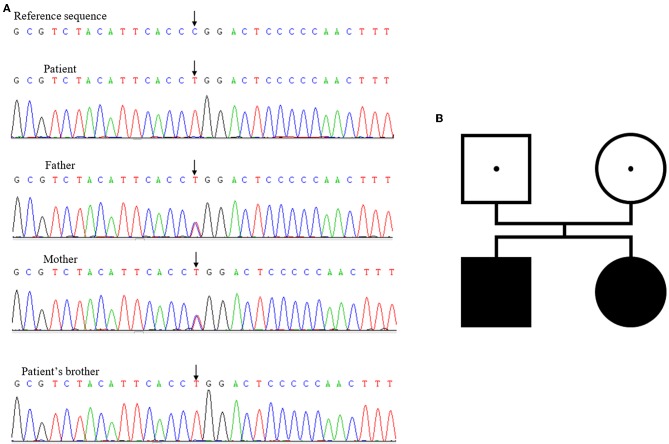
**(A)** Mutations of *TBX19* detected in family members. **(B)** The family genetic map.

Initially, next-generation sequencing for the brother suggested that no pathogenic mutations existed. When his sister suffered from similar symptoms, she underwent a Sanger sequencing together with her parents, revealing a homozygous mutation of *TBX19* gene, C.377 (exon2) C>T, p. P126L. Both of her parents had heterozygous mutations at the same locus. We reevaluated the brother's gene sequencing results, which were the same as his sister (Figure 1).

To date, this genetic mutation has not been reported on OMIM, HGMD, or Clinvar databases. This was a missense mutation, and according to the protein structure prediction, it was highly suspected that the altered protein structure may have been pathogenic. Ultimately, after a comprehensive analysis of the clinical features, effective treatment with glucocorticoid therapy, and genetic testing, our diagnosis was confirmed as CIAD.

## Discussion

Isolated ACTH deficiency is a rare disease that was first described by Steinberg in 1954 (2). The disease presents itself as a pituitary disorder characterized by a reduction in the secretion of ACTH. According to a recent epidemiological investigation in Japan, the prevalence of AIAD is 3.8–7.3 per 100,000 people (3). Globally, there is a lack of epidemiological information regarding CIAD. This disease is documented mostly in case reports or series. If pediatricians fail to recognize this disease in the neonatal period, it can result in 20–25% mortality (4, 5).

The symptoms lack specificity on the onset of disease manifestation, making it difficult to recognize early. Akcan et al. reported on a 7-years-old boy who was diagnosed with CIAD, presenting a recurrent respiratory tract infection (6). Pham et al. summarized eight case series on neonatal and late-onset forms of CIAD (7). The clinical manifestations of neonatal CIAD were seizures with hypoglycemia, major hypothermia, fever, and shock. The clinical manifestations of late-onset CIAD were seizures and hypoglycemia. Another typical sign of CIAD is prolonged cholestatic jaundice (8). Atypical manifestations include vomiting, abdominal pain, asthenia, and neuropsychiatric symptoms, such as irritability, difficulty with physical activities, and anorexia. In our case report, the patient and her brother had symptoms of hypoglycemia and progressive cholestatic jaundice. The severe hypoglycemia caused seizures, and the brother suffered from irreversible brain damage, which manifested as intractable epilepsy and permanent developmental delay. In summary, pediatricians should recognize the early onset of cholestasis, hypoglycemia, and seizures, as the triad of symptoms are highly indicative of CIAD.

The detailed pathophysiologic mechanisms behind CIAD have not been thoroughly studied. ACTH is the upstream hormonal regulator of the hypothalamic-pituitary-adrenal axis, and the hyposecretion of ACTH eventually leads to a lack of cortisol. However, the reasons for liver function damage and cholestasis remain unclear. Rose et al. (9) used liver-specific GR-knockout mice to demonstrate the key role of glucocorticoids in the regulation of bile acid (9). Normal levels of glucocorticoid combine with the glucocorticoid receptor (GR) in the liver, and consequently, help maintain homeostasis in the body. Cortisol deficiency may decrease the efficiency of bile acid transport. As clearance of bile acid declines while bile acid aggregates in hepatocytes, biliary ducts, and blood, it penetrates the blood-brain-barrier. This reduces corticotropin-releasing hormone (CRH). Reduced CRH suppresses the hypothalamic-pituitary-adrenal axis and causes an imbalance in bile acid metabolism, resulting in a vicious cycle.

According to the whole exome sequencing result, genetic analysis of the *TBX19* gene was detected. The patient and her brother were both homozygous for the c.377 (exon2) C>T. *TBX19 (Tpit)* gene located in 1q24.2 and encoded for the protein: T-box transcription factor 19 (10). This transcription factor is characterized by a highly conserved DNA-binding motif (T-box) and involved the regulation of developmental processes. In this case, both of the probands were inherited their parents' abnormal gene and suffered from CIAD. The mutation site has not been published before on OMIM, HGMD, or Clinvar database.

In addition, the presentation of CIAD is similar to the presentation of congenital hypopituitarism, with multiple anterior pituitary hormone deficiencies (11). It requires neuroimaging and selection of hormone assays to establish deficiency of growth hormone. This is with or without insufficiency of other pituitary hormones (12). The finding of jaundice with direct hyperbilirubinemia is well-known in the pediatric endocrine literature on congenital hypopituitarism, an entity which is common and should be considered in the differential diagnosis.

Although CIAD may have an unfavorable prognosis, timely glucocorticoid treatment is vital to lower the risk of recurrent hypoglycemia and uncontrolled epilepsy. As previously reported, hydrocortisone 15 mg/m^2^/day is effective (13). Replacement with glucocorticoid therapy at maintenance restores normoglycemia and prevents further hypoglycemic seizures. Continued follow-up is needed to assess the long-term outcome and prognosis of CIAD.

## Data Availability Statement

All data included in this study is available. If readers want to learn more, he/she can contact the corresponding authors.

## Ethics Statement

Written informed consent was obtained from the individual(s), and minor(s)' legal guardian/next of kin, for the publication of any potentially identifiable images or data included in this article.

## Author Contributions

CP wrote the manuscript. GS collected the data and followed up the case. ZT and XH supervised the management and follow up of the case. All the authors revised and approved the final manuscript and agreed to be accountable for the content of the work.

### Conflict of Interest

The authors declare that the research was conducted in the absence of any commercial or financial relationships that could be construed as a potential conflict of interest.
